# Evaluating the knowledge of stroke management among the non-neurological healthcare professionals in an underdeveloped county in Southwestern China

**DOI:** 10.1371/journal.pone.0351499

**Published:** 2026-06-17

**Authors:** Xia Huang, Yuanbin Zhao, Shiquan Wang, Chao Lin, Li Xie, Ping Li, Xilin Liu, Zihan He, Lili Yang

**Affiliations:** 1 Department of Neurology, Tongjiang County People’s Hospital, Bazhong, China; 2 Department of Science and Education, Tongjiang County People’s Hospital, Bazhong, China; 3 Department of Orthopaedics, Sichuan Provincial People’s Hospital, School of Medicine, University of Electronic Science and Technology of China, Chengdu, China; 4 Department of Neurology, Sichuan Academy of Medical Sciences & Sichuan Provincial People’s Hospital, University of Electronic Science and Technology of China, Chengdu, China; King Saud University Medical City, SAUDI ARABIA

## Abstract

**Objective:**

This study aims to investigate the knowledge of stroke management among non-neurological healthcare professionals (HCPs) in an underdeveloped county in western China.

**Methods:**

The study proceeded in two phases. First, the Acute Stroke Management Questionnaire (ASMaQ) underwent a systematic intercultural adaptation into Chinese, followed by an evaluation of its psychometric properties. Second, a cross‑sectional survey using the adapted Chinese ASMaQ was administered to non‑neurological HCPs working in an underdeveloped county in southwestern China. A convenience sampling method was used in the recruitment. Demographic information, including age, gender, years of service, job position (doctor/nurse), department, professional level, medical facility level (comprehensive hospital/noncomprehensive medical facility), and whether the participant had a neurologist partner or not during routine practice, was collected. The internal consistency was then measured with Cronbach’s α. Further statistical analyses were conducted to identify potential influencing factors of the ASMaQ score.

**Results:**

After a standardized intercultural adaptation and evaluation process, a reliable localized Chinese version of ASMaQ was obtained, with an overall Cronbach’s α value of 0.77. A total of 252 HCPs completed the questionnaires, and 225 qualified questionnaires were included in the statistical analysis. The results revealed that the rate of good performance of overall ASMaQ among non-neurological HCPs was 69.4%. For the three subscales of ASMaQ, the rates of good performance for general stroke knowledge (GSK), hyperacute stroke management (HSM), and advanced stroke management (ASM) were 73.3%, 64.7%, and 70.1%, respectively. Furthermore, we found that the factors influencing the overall ASMaQ score were primarily gender, job position, medical facility level, and with/without neurologist partners. Specifically, females performed worse than males did (106.7 ± 7.7 vs. 110 ± 8.1; P = 0.003), nurses performed worse than doctors did (106.3 ± 7.6 vs. 110.0 ± 7.9; P < 0.001), the HCPs in noncomprehensive medical facilities performed worse than those in comprehensive hospitals did (105.2 ± 9.3 vs. 108.5 ± 7.3; P = 0.02), and the HCPs without neurologist partner in the routine clinical practise performed worse than those with (106.1 ± 8.1 vs 109 ± 7.5, P = 0.005). Multiple regression analysis revealed that the job position and the level of medical facility were the independent influencing factors for the total ASMaQ score (P < 0.001).

**Conclusion:**

The Chinese version of ASMaQ comprehensively assesses the understanding of stroke management knowledge among HCPs. This study reflected the lack of stroke management knowledge among non-neurological HCPs in underdeveloped western regions of China for the first time and revealed that nurses and HCPs in noncomprehensive medical facilities were more likely to have insufficient knowledge of stroke management. Strengthening their training will significantly help improve stroke treatment rates, thereby reducing the burden of stroke-related morbidity and mortality.

## Introduction

The 2021 Global Burden Disease study reported that China had approximately 20.80 million cases of ischemic stroke [1]. Stroke is the third leading cause of death and the primary cause of disability among Chinese residents [2,3], imposing substantial socioeconomic burdens. The incidence, prevalence, and mortality of stroke in China exhibit a distinct geographical disparities, with the southwestern provinces showing the highest age-standardized mortality rate at 508.8 per 100,000 population [4]. Moreover, according to the *China Health Statistics Yearbook 2023*, the prevalence and mortality of stroke are higher in rural areas than in urban areas [4,5].

China began developing a stroke prevention and treatment system several decades ago [6]. Although the establishment of stroke centers has improved in-hospital stroke treatment capabilities, treatment delays remain a major challenge. A recent report revealed that only 11.79% of patients with acute ischemic stroke (AIS) arrive at the hospital within 3 hours of symptom onset, whereas 51% arrive within 24 hours, indicating substantial prehospital barriers to timely reperfusion therapy [4]. This situation is likely inevitably more severe in rural areas of Southwestern China because of economic and transportation constraints. Residents in rural areas may initially seek care at medical institutions without stroke units, such as community hospitals or clinics, which further challenges the ability of healthcare professionals (HCPs) to promptly identify and refer patients with stroke. Therefore, improving the stroke treatment efficiency in rural areas of southwestern China depends not only on the availability of stroke units but also on the stroke-related knowledge of grassroot HCPs [7].

Moreover, in-hospital strokes account for a substantial proportion of all stroke cases (6.5–15%) [8], and are generally more severe and associated with poorer outcomes than community-onset strokes [9–11]. A key strategy for improving the prognosis of patients with in-hospital stroke is reducing delays in reperfusion therapy [12]. Unfortunately, most patients with in-hospital stroke are not identified promptly [12,13]. Studies have revealed that only 13–52% of patients with in-hospital stroke received evaluation within 3 hours of symptom onset [14,15]. The delay in treatment may be attributed to three main factors: First, patients often lack continuous monitoring, and symptoms are typically detected only during routine ward rounds [16]; second, nonneurological HCPs may exhibit cognitive bias and reduced sensitivity to neurological deficits; even when symptoms are recognized, they may be erroneously attributed to postanesthesia effects or other drug side effects [8,17]; and third, some HCPs may have limited awareness of current advances in stroke treatment, leading to the misconception that no effective therapies are available, resulting in delayed notification to stroke teams [12]. Thus, the successful treatment of in-hospital stroke depends largely on HCPs’ ability to recognize stroke symptoms and their awareness of available treatment options [18].

Accordingly, understanding the stroke-related knowledge of HCPs, especially non-neurologists, is essential. In this study, we conducted a survey assessing stroke management knowledge among non-neurological HCPs in an underdeveloped county located in Southwestern China, with an aim to evaluate stroke awareness among grassroots non-neurological HCPs and to identify potential intervention directions for improving stroke treatment capacity in underdeveloped areas and increasing the in-hospital stroke treatment rate.

## Materials and methods

### Study design

This study was completed from July 1st to December 10th 2025 and proceeded in two phases. First, the Acute Stroke Management Questionnaire (ASMaQ) underwent a systematic intercultural adaptation into Chinese, followed by an evaluation of its psychometric properties. Second, a cross-sectional online questionnaire study was conducted among HCPs throughout Tongjiang County, an underdeveloped county in southwestern China. HCPs in this study included doctors, assistant doctors, and nurses from all the clinical departments; while the personnel from administrative, logistics, and auxiliary departments such as radiology, laboratory, nuclear medicine, and transfusion departments were not included.

### Setting

Tongjiang County, Sichuan Province is located in western China (north latitude 31°39’-32°034’, east longitude 106°59’-107°45’). According to the 2024 Statistical Bulletin on National Economic and Social Development of Tongjiang County [19], the county has 13 comprehensive or specialized hospitals, 32 township health clinics, 5 community service stations, and 452 village clinics. Only two comprehensive hospitals have stroke units that can routinely perform thrombolysis and thrombectomy treatments.

### Instrument

The online questionnaire consisted of two parts. In Part 1 (demographic information), data on age, gender, education level, years of work experience, job position (doctor/nurse), department, professional level, medical facility level (comprehensive hospital/noncomprehensive medical facility), and the presence or absence of a neurologist partner during routine practice were collected.

For departments, we have categorized them into five classifications: internal medicine (including cardiology, respiratory, endocrinology, nephrology, oncology, hematology, rheumatology and immunology, traditional Chinese medicine, and rehabilitation, but excluding neurology), surgery (including general surgery, thoracic surgery, urology, neurosurgery, vascular surgery, and orthopedics), emergency and intensive critical care, others (including pediatrics, obstetrics and gynecology, dermatology, psychiatry, dentistry, ophthalmology, and otolaryngology), and undifferentiated departments. A comprehensive hospital referred to a secondary or tertiary hospital that was equipped with at least internal medicine, surgery, obstetrics and gynecology, pediatrics, and emergency departments. A noncomprehensive medical facility referred to primary healthcare institutions (such as township health clinics) or specialized hospitals (such as maternal and child health hospitals). A neurologist partner was defined as the facility of the HCPs had neurologists on staff, and meanwhile, they had direct access to neurology consultation or participated in joint rounds or case discussions with neurologists in their routine practice. Part 2 was the Chinese version of the ASMaQ.

The ASMaQ scale was developed by Sim et al. [18] to assess HCP knowledge levels regarding acute stroke management. The scale comprises 29 items divided into three subscales: general stroke knowledge (GSK) (Items 1–10), hyperacute stroke management (HSM) (Items 11–19), and advanced stroke management (ASM) (Items 20–29). The questionnaire uses a 5-point Likert scale for scoring, with 7 items employing reverse scoring (Items 8–11, 17, 18, and 29). The total score ranges from 29 to 145, with higher scores closer to 145 indicating better stroke management knowledge. The total score of GSK and ASM ranges from 10 to 50, while 9–45 for HSM. The Cronbach’s α coefficient for the questionnaire is 0.82 [18]. The questionnaire has preliminarily been applied in several countries [20–23], but there were no relevant studies regarding Chinese HCPs.

To ensure data quality, the questionnaire included an attention‑check item (‘Please select “apple” from the following options: apple, grape, banana’). Participants who failed this item were considered inattentive responders and were excluded from subsequent analyses.

#### Intercultural adaptation and evaluation of the ASMaQ.

This process involved the translation and retranslation of ASMaQ, expert review, and preliminary application [24].

1) Translation and Retranslation of ASMaQ

Two independent translators separately translated the questionnaire into Chinese. The researchers consolidated the translations into a single document. The questionnaire was subsequently retranslated into English by two different professional translators who are proficient in both Chinese and English. The research team evaluated and discussed the document, resulting in the initial draft of the Chinese version of ASMaQ.

2) Expert review

In terms of content validity, the obtained Chinese version and the original English version were submitted to eleven specialists (5 neurology doctors, 2 neurology nurses, 2 emergency doctors, and 2 emergency nurses) working in different specialties related to the subject. The experts were asked to grade the questionnaire items in terms of their relationships with Chinese culture, comprehensibility, and extensiveness on a four-point scale (1. Not appropriate, 2. The item needs improvement to be appropriate; 3. Appropriate but needs minor changes, and 4. Very appropriate) and offer a suggestion, if possible.

For all 29 items, eleven experts rated them 1 or 2 points (as shown in S1 Table). None of the experts gave a negative answer for any item. In line with expert opinions, changes were made to items ASM-2, 4, 5, and 9. Item ASM-2, “Are you familiar with FAST (Face, Arm, Speech, Time)?,” was modified to “Are you familiar with FAST (Face, Arm, Speech, Time) or Stroke 120?” (in Chinese), since the “Stroke 120” mnemonic is a more localized and widely promoted mnemonic for recognizing the symptoms of strokes in China [25]. In items ASM-4 and ASM-5, “机械取栓术” (a more concise translation term of mechanical thrombectomy treatment, also more commonly used) has been suggested to replace the “机械血栓清除术”(the literal translation of Mechanical thrombectomy treatment) in the first version. For item ASM-9, “治疗时间窗” (the literal translation of ‘therapeutic time window’) was suggested to replace “治疗窗” (the literal translation of ‘therapeutic window’) in the first version. These changes were made to obtain appropriate items in terms of clarity and comprehensibility. The final Chinese version of the ASMaQ questionnaire and its instructions are provided in S2 Table.

3) Preliminary Applications

To ensure that the questionnaire items were easily and readily comprehensible, a pilot study was conducted with 14 HCPs (6 doctors and 8 nurses). Participants found the questionnaire culturally appropriate, comprehensible, and free of ambiguity (the results are available in Supporting Information-Raw Data Sheet 2). Consequently, no modifications were needed. These individuals were excluded from the main analysis.

### Sample size calculation

The sample size calculation was performed using PASS 2023 (Power Analysis and Sample Size Software), Version 23.0.2 (NCSS, LLC., Kaysville, Utah, USA, ncss.com/software/pass). According to the 2024 report of Tongjiang County, the county included 3,699 (N) healthcare workers. The confidence level was 0.95 (α = 0.05, Zα_/2_ = 1.96), and the standard deviation was assumed to be 9.29 (E) [21]. To produce a confidence interval with a distance of no more than 1.86 (σ) from the sample mean to either limit, a sample of 94 individuals from the population was needed [26]. The formula for sample size calculation is:


$$\begin{array}{c} n=\frac{{\left(\frac{Z_{\alpha /\text{2}}\times \sigma }{E}\right)}^{\text{2}}}{\text{1}+\frac{{\left(\frac{Z_{\alpha /\text{2}}\times \sigma }{E}\right)}^{\text{2}}}{N}} \end{array}$$

In anticipation of a 20% dropout rate, at least 118 subjects needed to be enrolled.

### Data collection

A convenience sampling method was used to recruit participants. With the assistance of the Tongjiang Health Administration Department, we have sent emails to all the medical institution management departments in this county, explaining the purpose and procedures of this survey research. We request that they post promotional posters on their institution’s bulletin boards, which include QR codes for completing the online questionnaire. Interested HCPs may participate in the survey by scanning the QR code via WeChat on a voluntary basis. The interested participants were informed to complete the online questionnaire.

A total of 252 questionnaires were collected, 2 of which lacked signed informed consent; 7 were from HCPs in neurology, administrative, or auxiliary departments; and 18 contained trap questions with incorrect answers, all of which were excluded. Ultimately, 225 valid questionnaires were retained for analysis.

### Ethical approval

This study was approved by the Ethics Committee of Sichuan Provincial People’s Hospital and Tongjiang County People’s Hospital. Prior to data collection, participants were provided with an online-written informed consent form, where they could freely choose to agree or decline. Those who provided online-written consent completed the questionnaire.

### Statistical analysis

For data evaluation, categorical variables are described using numerical values and percentages, while quantitative data are presented as means and standard deviations (SDs). For all of the quantitative data, appropriate statistical methods were chosen on the basis of normality, which was checked with the Kolmogorov‒Smirnov test.

In evaluating ASMaQ scores, we first defined the desired response (scores 4 and 5) as good knowledge and the undesired response (scores 1 and 2) and neutral response (score 3) as poor knowledge on the basis of previous research approaches and the opinions of stroke neuroscientists [21]. We estimated the proportions of healthcare professionals with good and poor knowledge of stroke using 95% confidence intervals (CIs).

When evaluating the relationship between the variables and ASMaQ scores, Pearson’s correlation analysis or nonparametric Spearman’s ranked correlation analysis were conducted to explore the relationships between the ASMaQ score and subscale scores and the independent variables, including age and working years, according to the normality distribution.

Unpaired t tests or Mann‒Whitney tests were used to determine whether the ASMaQ score and subscale scores differed among groups according to gender, job position, medical facility level, and the presence or absence of a neurologist partner, according to the normality distribution. One-way ANOVA or were chosen to determine the differences in the ASMaQ score and subscale scores among groups distributed by education level, professional level, and department, according to the normality distribution.

Multiple linear regression was used to further assess the independent factors of the ASMaQ score/subscale scores (by verification of the normality of the residuals). Age, education level, working years, job position, professional level, department, level of medical facility, and having a neurologist partner were included as possible independent variables for the multiple linear regression model. P < 0.05 was considered to indicate statistical significance.

The Cronbach’s α index for internal consistency of the questionnaire was analyzed using IBM SPSS Statistics 21. Previous literature indicates that a Cronbach’s α coefficient of 0.70 is considered adequate for internal consistency analysis [27]. Other statistical analyses were performed using GraphPad Prism v8.0.2 software.

## Results

### Reliability of the Chinese version of ASMaQ

Content validity was assessed by 11 expert opinions. In this study, the content validity index value was determined to be 0.99. The overall Cronbach’s α coefficient in this study was 0.77.

### Demographic characteristics

A total of 225 questionnaires were included in the analysis. The detailed demographic characteristics is displayed in Table 1.

**Table 1:** The demographic characteristics of the 225 participants.

Characteristic	Value
Gender (F/M), N (%)	159/66 (70.6%/29.3%)
Age (years), mean [SD] (range)	35.8 ± 9.6 (21-59)
Education Level	
Master/doctor, N (%)	4 (1.7%)
Bachelor, N(%)	129 (57.3%)
Associate college, N (%)	81 (36.0%)
Senior high school, N (%)	11 (4.8%)
Job position	
Doctor, N (%)	86 (38.2%)
Nurse, N(%)	139 (61.7%)
Working years (years), mean [SD] (range)	12.9 ± 9.7 (0-40)
Professional level	
Not yet rated, N (%)	27 (12.0%)
Junior, N(%)	97 (43.1%)
Intermediate, N (%)	79 (35.1%)
Senior, N (%)	22 (9.7%)
Department	
Internal medicine, N (%)	66 (29.3%)
Surgery, N(%)	65 (28.8%)
Emergency/intensive critical care, N (%)	30 (13.3%)
Others, N (%)	6 (2.6%)
Undifferentiated department, N (%)	58 (25.7%)
Medical facility level	
Comprehensive hospital, N (%)	168 (74.7%)
Non-comprehensive medical facilities, N (%)	57 (25.3%)
With neurologists partner in the routine clinical practise	
Yes, N (%)	122 (54.2%)
No, N (%)	103 (45.7%)

### Knowledge of stroke management

**Fig 1:**
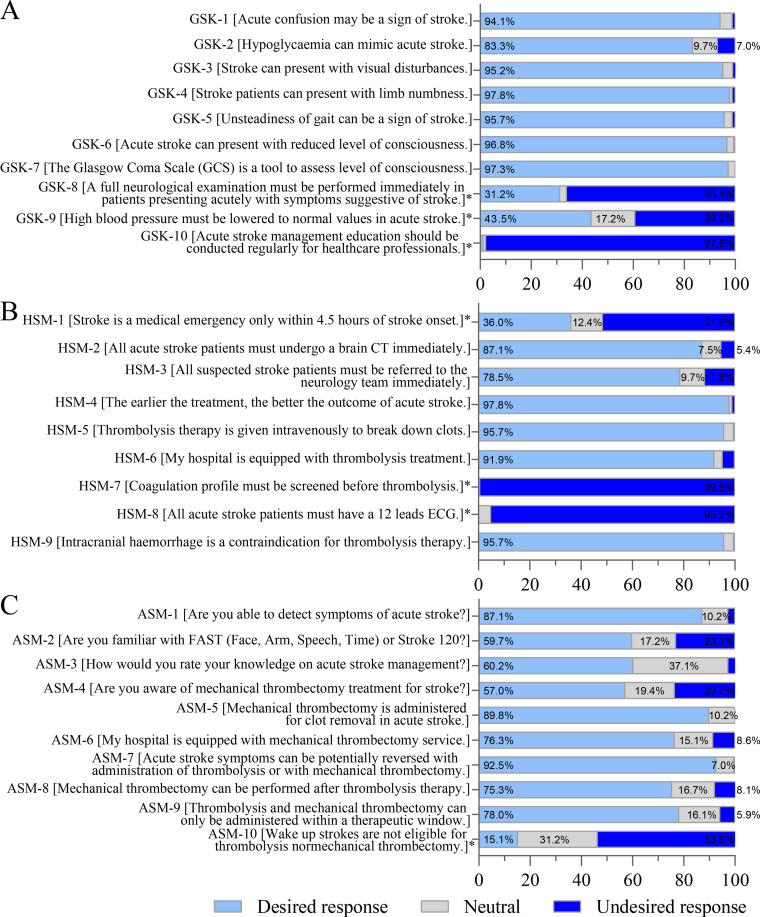
Participants’ response to ASMaQ. **A)** General Stroke Knowledge; **B)** Hyperacute Stroke Management; **C)** Advanced Stroke Management. An asterisk (*) indicates a negative answer is the desired response. The proportions which are less than 5% are not shown in the figure.

The mean ASMaQ total score was 107.7 ± 7.9, with subscale means of 37.8 ± 2.9 for GSK, 31.7 ± 2.7 for ASM, and 38.3 ± 5.1 for HSM. Overall, 69.4% of non neurologist HCPs demonstrated good stroke related knowledge (95% CI: 57.0–81.7%). Subscale performance showed a similar pattern: GSK had the highest proportion of adequate knowledge (73.7%, 95% CI: 48.4–99.0%), followed by ASM (70.1%, 95% CI: 54.5–85.8%) and HSM (63.7%, 95% CI: 32.3–95.1%). The distribution of desired, neutral, and undesired responses is presented in Fig 1 and S3 Table.

The survey revealed that for GSK, the highest percentage of undesired response items was GSK-10 (Acute stroke management education should be conducted regularly for healthcare professionals, 97.3%), followed by GSK-8 (A full neurological examination must be performed immediately in patients presenting acutely with symptoms suggestive of stroke, 66.7%) and GSK-9 (High blood pressure must be lowered to normal values in acute stroke, 41.3%). Regarding HSM, the proportions of undesired responses were high for HSM-7 (Coagulation profile must be screened before thrombolysis, 99.1%) and HSM-8 (All acute stroke patients must have a 12 leads ECG, 94.7%), followed by HSM-1 (Stroke is a medical emergency only within 4.5 hours of stroke onset, 55.6%). Among the ASM items, ASM-10 had the highest proportion of undesired responses (Wake up strokes are not eligible for thrombolysis nor mechanical thrombectomy, 54.7%), followed by ASM-4 (Are you aware of mechanical thrombectomy treatment for stroke, 22.2%) and ASM-2 (Are you familiar with FAST or Stroke 120, 21.8%). The detailed information is presented in Fig 1.

### Factors correlated with stroke knowledge

To further investigate the factors correlated with the knowledge of stroke management, we first evaluated whether age and working years were associated with the total ASMaQ score and its subscale scores. The statistical correlations only existed between age and ASM scores (P = 0.01, r = 0.17) (as shown in S4 Table).

**Fig 2:**
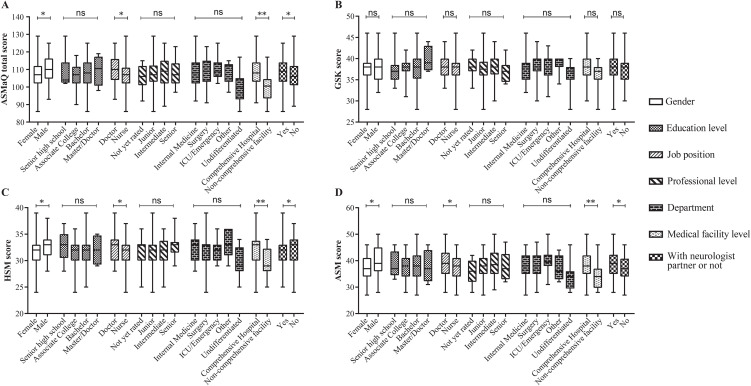
The differences of the total ASMaQ score and subscale scores among participants’ subgroups distributed by gender, education level, job position, professional level, department, medical facility level, and presence of a neurologist partner or not. GSK, general stroke knowledge; HSM, hyperacute stroke management; ASM, advanced stroke management; Ns, not significant; * P < 0.05; ** P < 0.001.

Second, we analyzed whether the total ASMaQ score and subscale scores differed among participant subgroups according to gender, education level, job position, professional level, department, medical facility level, and presence of a neurologist partner. We found that the factors influencing the overall ASMaQ score were primarily gender, job position, professional level, and medical facility level. Specifically, females performed worse than males did (106.7 ± 7.7 vs. 110 ± 8.1; P = 0.003), nurses performed worse than doctors did (106.3 ± 7.6 vs. 110.0 ± 7.9; P < 0.001), the HCPs in noncomprehensive medical facilities performed worse than those in comprehensive hospitals did (105.2 ± 9.3 vs. 108.5 ± 7.3; P = 0.02), and the HCPs without neurologist partner in the routine clinical practise performed worse than those with (106.1 ± 8.1 vs 109 ± 7.5, P = 0.005) (as shown in Fig 2 and S5 Table).

Regarding the three subscales, no factors were found to influence the GSK score. Female participants (P = 0.001), nurses (P = 0.002), and participants in noncomprehensive medical facilities (P < 0.001) were similarly more likely to have worse HSM scores. In addition to female participants (P < 0.001), nurses (P = 0.001), and participants working in noncomprehensive medical facilities (P < 0.001), the professional levels (P = 0.02), and having a neurologist partner in routine clinical practice (P = 0.01) were also significant influencing factors for the ASM score.

### Multivariate linear regression of the ASMaQ score

To identify the independent influencing factors for ASMaQ and its subscale scores, we conducted a multivariate linear analysis (the results are displayed in Table 2). The results of the analysis revealed that job position and the level of medical facility were the independent predictors of poorer overall ASMaQ and HSM subscores among non-neurological HCPs. With neurologist partner was the only independent influencing factor for the ASM score (P = 0.03).

**Table 2:** Multivariable linear regression of the ASMaQ score/subscale scores in healthcare professionals.

Variables	B	S.E.	95%CI	*P*
**Total score**				
Job position	−3.52	1.55	−6.65 to −0.46	0.02
Medical facility level	−4.51	2.16	−8.79 to −0.25	0.04
**General Stroke Knowledge**				
No variable	–	–	–	–
**Hyperacute Stroke Management**				
Job position	−1.08	0.51	−2.10 to −0.07	0.04
Medical facility level	−2.75	0.72	−4.16 to −1.34	<0.001
**Advanced Stroke Management**				
With neurologist partner in the routine clinical practice	−1.52	0.71	−2.91 to −0.13	0.03

## Discussion

In this study, the Chinese version of the ASMaQ was established for the first time, and its consistency was validated. Furthermore, a cross‑sectional survey was conducted among non-neurological HCPs in an underdeveloped county in Southwestern China, revealing that the rate of good performance in ASMaQ was 69.4%. Our study demonstrated that nurses and medical facility levels were the independent influencing factors for participants’ overall stroke knowledge. This study provides a Chinese version of the survey questionnaire for investigating stroke management knowledge among non-neurological HCPs in China and, for the first time, applies this questionnaire to identify deficiencies in stroke knowledge among HCPs in underdeveloped regions of Southwestern China, thereby providing a basis for the subsequent targeted development of stroke knowledge training programs.

This study revealed that the knowledge of essential thrombolytic examinations is limited among non-neurological HCPs. First, 66.7% of non-neurological HCPs mistakenly believed that a comprehensive neurological examination was necessary. However, the National Institutes of Health Stroke Scale (NIHSS) is sufficient for the preliminary assessment of patients with acute stroke [28], whereas a comprehensive neurological examination may lead to delayed management. Second, most non-neurological HCPs incorrectly believed that “the coagulation profile must be screened before thrombolysis” and “all acute stroke patients must undergo a 12-lead ECG.” TheAmerican Heart Association (AHA)/American Stroke Association (ASA) guidelines recommend that all patients only need to have their blood glucose measured before intravenous thrombolysis [29]. Owing to the low probability of platelet abnormalities and coagulation abnormalities in the population, intravenous thrombolysis should not be delayed by awaiting laboratory results for platelet or coagulation test in patients without a previous history of related diseases or a significant bleeding tendency [30–32]. Similarly, although ECG is useful for evaluating the etiology of cardiogenic embolism, it is not a mandatory examination before thrombolysis. Previous surveys conducted in other countries reported similar findings [20,21], suggesting that HCPs share similar misconceptions, which is a key point in stroke training.

Furthermore, non-neurological HCPs demonstrated misconceptions regarding blood pressure management in AIS patients. Approximately 40% of non-neurological HCPs believed that blood pressure should be reduced to normal levels. The Chinese Stroke Guidelines stipulate that antihypertensive drugs should be used with caution after AIS within 24 hours [33]. Excessive or drastic reductions in blood pressure may exacerbate cerebral hypoperfusion and trigger stroke progression [33]. However, excessive blood pressure reduction is frequently, observed in clinical practice. One study reported that 62.6% of patients with stroke received excessive antihypertensive drugs [34]. In another study targeting general practitioners, 27% desired a significant reduction in blood pressure, whereas 14% were uncertain regarding the ideal blood pressure target [35]. Similar findings have also been explored in surveys in other countries [20,21], suggesting that blood pressure management should be repeatedly and emphatically emphasized during stroke management education and training.

Although Chinese non-neurological HCPs were generally aware of intravenous thrombolysis (94.7%), a proportion higher than that reported in Nigeria (79.2%) [20], knowledge of advanced stroke management was limited. For example, 55.6% of participants believed that stroke was a medical emergency only within 4.5 hours after the onset of stroke; 54.7% considered thrombolysis or mechanical thrombectomy unsuitable for wake-up strokes, and 28% expressed uncertainty. Moreover, only 60.4% of non-neurological HCPs reported familiarity with mechanical thrombectomy. These findings align with those of previous studies [20,21], and further highlight inadequate awareness among non-neurological HCPs regarding mechanical thrombectomy and its optimal time window, as well as the beyond-time-window thrombolysis under imaging guidance. Consequently, potentially treatable patients with in-hospital stroke may be missed because of a lack of knowledge [12]. Previous in-hospital stroke studies have shown that patients with in-hospital stroke are more likely to have contraindications to thrombolysis, such as comorbid surgeries or anticoagulant use, resulting in significantly lower thrombolysis rates in these patients than in patients with community-onset stroke [36]. Therefore, enhancing training and updating the HCPs’ knowledge regarding advanced stroke therapy is crucial to avoid iatrogenic treatment delays.

Our findings suggested that more attention should be given to the training about stroke management knowledge for HCPs in non-comprehensive medical facilities and nurses. Importantly, compared with other team members, nurses are more likely to be the first to identify in-hospital acute stroke [12], which may be attributed to their greater time spent at the patient’s bedside [23,37–39]. Many in-hospital stroke management protocols therefore emphasize nurse-led stroke management processes [13]. A previous ASMaQ survey conducted specifically among nurses revealed that different teaching methods for training and reinforcement learning are effective at improving nurses’ stroke-related knowledge. Nurse administrators and educators may also use the ASMaQ to assess baseline stroke knowledge before employment, thereby creating customized stroke-oriented education programs [23].

As official China stroke reports have revealed the southwestern provinces and rural area have higher stroke mortality rates [4,5], strategies for improving the efficiency of stroke treatment in these area are urgently needed. This study was conducted in Tongjiang County, which is located in Southwestern China and has a relatively underdeveloped economy. The county covers an area of approximately 4120 km^2^ and is mostly mountainous, with limited transportation infrastructure. Of the approximately 502 medical institutions or clinics in the county, only two of which are comprehensive hospitals equipped with stroke units. Therefore, the survey in this county can to some extent reflect the current situation of rural areas in Southwestern China. In these areas, most patients with stroke initially seek care at nearby noncomprehensive medical facilities, requiring the HCPs to promptly identify stroke symptoms and refer them to hospitals with stroke units. Our findings demonstrated that HCPs in noncomprehensive medical facilities have significantly less knowledge about stroke. This cognitive bias among HCPs may contribute to missed therapeutic windows in patients who could still benefit from timely intervention. Therefore, we urge that the promotion of stroke knowledge among HCPs in noncomprehensive medical facilities should be prioritized by health management authorities, as this would significantly increase stroke treatment rates in underdeveloped regions and alleviate the burden on society and families.

Moreover, our study identified several knowledge gaps in stroke management among non-neurological HCPs, many of which were particularly relevant to in-hospital strokes. For example, the misconceptions regarding mandatory coagulation/ECG testing were found to be common in non-neurological HCPs, which directly impacts in-hospital strokes, where delays may be compounded by unnecessary waiting for the tests; second, the lack of awareness about wake-up stroke treatment was particularly relevant to in-hospital strokes, as these strokes often occur overnight or in unmonitored settings; and third, knowledge gaps among nurses are critical given their role as first detectors of in-hospital strokes. Targeted interventions addressing these deficiencies may contribute to improving the rate of timely in-hospital stroke treatment.

### Limitations

This study has several limitations including recall bias from respondents, which may not reflect their actual knowledge level. In this study, the use of convenience sampling may have introduced a certain degree of bias, such as insufficient representation of non-comprehensive hospitals. Similarly, as this study recruited interested HCPs by posting recruitment posters, the questionnaire response rate could not be determined, which may have compromised the assessment of representativeness and potential nonresponse bias. The HCPs who choose to respond may differ from non-respondents (potentially those with greater interest in stroke), which could lead to an overestimation of knowledge levels. The cross-sectional design helps to identify knowledge gaps but cannot assess the influence of these gaps on clinical outcomes. Furthermore, although the county-level research conducted at this institute can partially represent the grassroots conditions of Southwestern China, which are characterized by inconvenient transportation and underdeveloped economic conditions, caution is warranted when generalizing the findings to the entirety of southwestern China. Future studies require multicenter longitudinal observational research that links ASMaQ scores to clinical outcomes, such as door-to-needle times or thrombolysis rates.

## Conclusions

The localized version of ASMaQ can comprehensively assess Chinese HCP knowledge regarding stroke management with good consistency. The first survey in this study reflects the knowledge gaps in stroke among non-neurological HCPs in underdeveloped southwestern regions of China. To bridge this knowledge gap, it is crucial to regularly organize courses focused on stroke treatment. Moreover, HCPs in noncomprehensive medical facilities and nursing staff are more likely to have insufficient knowledge of stroke. However, given their critical role in disease detection and initiating stroke procedures, enhancing their training will significantly improve stroke treatment rates, thereby reducing the burden of stroke-related morbidity and mortality.

## Supporting information

S1 Table The expert review of the preliminary Chinese version of acute stroke management questionnaire. (DOCX)

S2 Table The Chinese version of acute stroke management questionnaire. (DOCX)

S3 Table Participants’ response to ASMaQ. (DOCX)

S4 Table The correlation between ASMaQ score/subscore and demographic variables in healthcare professionals. (DOCX)

S5 Table The comparisons of ASMaQ score/subscore among the healthcare professionals’ subgroups distributed by gender, education level, job position, professional level, department, medical facility level, and presence of a neurologist partner or not. (DOCX)

S1 File Supporting information-raw data. The raw data of the study.
(XLSX)
