# Supplementary material for: Evaluating the knowledge of stroke management among the non-neurological healthcare professionals in an underdeveloped county in Southwestern China
Source: PLoS One. 2026 Jun 17;21(6):e0351499. doi: 10.1371/journal.pone.0351499 (PMC13274870; doi:10.1371/journal.pone.0351499)
Supplement: S1 Table — (DOCX) [file pone.0351499.s001.docx]

**Supplementary Table S1. The expert review of the preliminary Chinese version of Acute Stroke Management Questionnaire.**

| ASMaQ | NO.1 | NO.2 | NO.3 | NO.4 | NO.5 | NO.6 | NO.7 | NO.8 | NO.9 | NO.10 | NO.11 |
| --- | --- | --- | --- | --- | --- | --- | --- | --- | --- | --- | --- |
| **General Stroke Knowledge** | | | | | | | | | | |  |
| GSK-1 | 4 | 4 | 4 | 4 | 4 | 4 | 4 | 4 | 4 | 4 | 4 |
| GSK-2 | 4 | 4 | 4 | 4 | 4 | 4 | 4 | 4 | 4 | 4 | 4 |
| GSK-3 | 4 | 4 | 4 | 4 | 4 | 4 | 4 | 4 | 4 | 4 | 4 |
| GSK-4 | 4 | 4 | 4 | 4 | 4 | 4 | 4 | 4 | 4 | 4 | 4 |
| GSK-5 | 4 | 4 | 4 | 4 | 4 | 4 | 4 | 4 | 4 | 4 | 4 |
| GSK-6 | 4 | 4 | 4 | 4 | 4 | 4 | 4 | 4 | 4 | 4 | 4 |
| GSK-7 | 4 | 4 | 4 | 4 | 4 | 4 | 4 | 4 | 4 | 4 | 4 |
| GSK-8 | 4 | 4 | 4 | 4 | 4 | 4 | 4 | 4 | 4 | 4 | 4 |
| GSK-9 | 4 | 4 | 4 | 4 | 4 | 4 | 4 | 4 | 4 | 4 | 4 |
| GSK-10 | 4 | 4 | 4 | 4 | 4 | 4 | 4 | 4 | 4 | 4 | 4 |
| **Hyperacute Stroke Management** | | | | | | | | | | |  |
| HSM-1 | 4 | 4 | 4 | 4 | 4 | 4 | 4 | 4 | 4 | 4 | 4 |
| HSM-2 | 4 | 4 | 4 | 4 | 4 | 4 | 4 | 4 | 4 | 4 | 4 |
| HSM-3 | 4 | 4 | 4 | 4 | 4 | 4 | 4 | 4 | 4 | 4 | 4 |
| HSM-4 | 4 | 4 | 4 | 4 | 4 | 4 | 4 | 4 | 4 | 4 | 4 |
| HSM-5 | 4 | 4 | 4 | 4 | 4 | 4 | 4 | 4 | 4 | 4 | 4 |
| HSM-6 | 4 | 4 | 4 | 4 | 4 | 4 | 4 | 4 | 4 | 4 | 4 |
| HSM-7 | 4 | 4 | 4 | 4 | 4 | 4 | 4 | 4 | 4 | 4 | 4 |
| HSM-8 | 4 | 4 | 4 | 4 | 4 | 4 | 4 | 4 | 4 | 4 | 4 |
| HSM-9 | 4 | 4 | 4 | 4 | 4 | 4 | 4 | 4 | 4 | 4 | 4 |
| **Advanced Stroke Management** | | | | | | | | | | |  |
| AMS-1 | 4 | 4 | 4 | 4 | 4 | 4 | 4 | 4 | 4 | 4 | 4 |
| AMS-2 | 4 | 2 | 3 | 4 | 4 | 4 | 2 | 4 | 4 | 4 | 4 |
| AMS-3 | 4 | 4 | 4 | 4 | 4 | 4 | 4 | 4 | 4 | 4 | 4 |
| AMS-4 | 4 | 3 | 4 | 4 | 3 | 4 | 3 | 4 | 4 | 4 | 4 |
| AMS-5 | 4 | 4 | 4 | 4 | 3 | 4 | 3 | 4 | 4 | 4 | 4 |
| AMS-6 | 4 | 4 | 4 | 4 | 4 | 4 | 4 | 4 | 4 | 4 | 4 |
| AMS-7 | 4 | 4 | 4 | 4 | 4 | 4 | 4 | 4 | 4 | 4 | 4 |
| AMS-8 | 4 | 4 | 4 | 4 | 4 | 4 | 4 | 4 | 4 | 4 | 4 |
| AMS-9 | 4 | 4 | 3 | 4 | 4 | 3 | 3 | 4 | 4 | 4 | 4 |
| AMS-10 | 4 | 4 | 4 | 4 | 4 | 4 | 4 | 4 | 4 | 4 | 4 |

In terms of content validity, the obtained Chinese version and the original English version were submitted to the opinion of eleven specialists (5 neurology doctors (Expert NO.1,3,4,5,6), 2 neurology nurses (Expert NO. 2, 7), 2 Emergency doctors (Expert NO. 10&11), and 2 emergency nurses (NO. 8&9)) working in different specialties related to the subject. The experts were asked to grade the questionnaire items in terms of their relationship with the Chinese culture, comprehensibility, and extensity according to a four-point scale (1. Not appropriate, 2.Item needs improvement to be appropriate, 3. Appropriate but needs little changes, and 4. Very appropriate) and offer a suggestion, if possible.

For all the 29 items, eleven experts gave the answer, “The item is very appropriate” or “the item is appropriate but needs little changes.” No expert gave a negative answer for the items. In line with expert opinions, changes were made to items AMS-2, 4, 5 and 9. Item AMS-2, “Are you familiar with FAST (Face, Arm, Speech, Time)?,” was modified to “Are you familiar with FAST (Face, Arm, Speech, Time) or Stroke 120?”(in Chinese), since the “Stroke 120” mnemonic is a more localized and widely promoted mnemonic for recognizing the symptoms of strokes. Item AMS-4 and ASM-5, the translation of “mechanical thrombectomy treatment” has been suggested as “机械取栓术”, instead of the “机械血栓清除术” in the first version. Item ASM-9, the translation of “a therapeutic window” has been suggested as “治疗时间窗”, instead of “治疗窗” in the first version. These changes were made to obtain appropriate items in terms of clarity and comprehensibility
