# Supplementary material for: Evaluating the knowledge of stroke management among the non-neurological healthcare professionals in an underdeveloped county in Southwestern China
Source: PLoS One. 2026 Jun 17;21(6):e0351499. doi: 10.1371/journal.pone.0351499 (PMC13274870; doi:10.1371/journal.pone.0351499)
Supplement: S2 Table — (DOCX) [file pone.0351499.s002.docx]

**Supplementary Table S2. the Chinese Version of Acute Stroke Management Questionnaire.**

| **The Chinese Version** | **The original English Version** |
| --- | --- |
| **卒中的一般知识** | **General stroke knowledge** |
| 1.急性意识模糊可能是卒中的征兆。  非常同意 //同意 //中立 //不同意 //非常不同意 | 1. Acute confusion may be a sign of stroke.   Strongly agree //Agree //Neutral //Disagree //Strongly disagree |
| 2.低血糖症可与急性卒中发病表现类似。  非常同意 //同意 //中立 //不同意 //非常不同意 | 1. Hypoglycaemia can mimic acute stroke.   Strongly agree //Agree //Neutral //Disagree //Strongly disagree |
| 3.卒中可引起视觉障碍。  非常同意 //同意 //中立 //不同意 //非常不同意 | 1. Stroke can present with visual disturbances.   Strongly agree //Agree //Neutral //Disagree //Strongly disagree |
| 4.卒中患者可能出现肢体麻木。  非常同意 //同意 //中立 //不同意 //非常不同意 | 1. Stroke patients can present with limb numbness.   Strongly agree //Agree //Neutral //Disagree //Strongly disagree |
| 5.突发步态不稳可能是卒中的征兆。  非常同意 //同意 //中立 //不同意 //非常不同意 | 1. Unsteadiness of gait can be a sign of stroke.   Strongly agree //Agree //Neutral //Disagree //Strongly disagree |
| 6.急性卒中可表现为意识水平降低。  非常同意 //同意 //中立 //不同意 //非常不同意 | 6.Acute stroke can present with reduced level of consciousness.  Strongly agree //Agree //Neutral //Disagree //Strongly disagree |
| 7.格拉斯哥昏迷量表是一种评估意识水平的工具。  非常同意 //同意 //中立 //不同意 //非常不同意 | 7. The Glasgow Coma Scale (GCS) is a tool to assess level of consciousness.  Strongly agree //Agree //Neutral //Disagree //Strongly disagree |
| 8.对突然出现提示卒中症状的患者，必须立即进行全面的神经系统检查。  非常同意 //同意 //中立 //不同意 //非常不同意 | 1. A full neurological examination must be performed immediately in patients presenting acutely with symptoms suggestive of stroke.   Strongly agree //Agree //Neutral //Disagree //Strongly disagree |
| 9.急性卒中时，升高的血压必须降低到正常值。  非常同意 //同意 //中立 //不同意 //非常不同意 | 1. High blood pressure must be lowered to normal values in acute stroke.   Strongly agree //Agree //Neutral //Disagree //Strongly disagree |
| 1. 应定期对医疗保健专业人员进行急性卒中管理教育。   非常同意 //同意 //中立 //不同意 //非常不同意 | 1. Acute stroke management education should be conducted regularly for healthcare professionals.   Strongly agree //Agree //Neutral //Disagree //Strongly disagree |
| **超急性期卒中管理** | **Hyperacute stroke management** |
| 1.卒中只有在发作的4.5小时内才属于医疗急症。  非常同意 //同意 //中立 //不同意 //非常不同意 | 1. Stroke is a medical emergency only within 4.5 hours of stroke onset.  Strongly agree //Agree //Neutral //Disagree //Strongly disagree |
| 2.所有急性卒中患者必须立即接受脑部CT检查。  非常同意 //同意 //中立 //不同意 //非常不同意 | 2. All acute stroke patients must undergo a brain CT immediately.  Strongly agree //Agree //Neutral //Disagree //Strongly disagree |
| 3.所有疑似卒中患者必须立即转诊到神经科团队。  非常同意 //同意 //中立 //不同意 //非常不同意 | 3. All suspected stroke patients must be referred to the neurology team immediately.  Strongly agree //Agree //Neutral //Disagree //Strongly disagree |
| 4.急性卒中治疗越早，预后越好。  非常同意 //同意 //中立 //不同意 //非常不同意 | 4. The earlier the treatment, the better the outcome of acute stroke.  Strongly agree //Agree //Neutral //Disagree //Strongly disagree |
| 5.溶栓治疗是通过静脉注射药物来分解血栓。  非常同意 //同意 //中立 //不同意 //非常不同意 | 5. Thrombolysis therapy is given intravenously to break down clots.  Strongly agree //Agree //Neutral //Disagree //Strongly disagree |
| 6.我院可进行静脉溶栓治疗。  非常同意 //同意 //中立 //不同意 //非常不同意 | 1. My hospital is equipped with thrombolysis treatment.   Strongly agree //Agree //Neutral //Disagree //Strongly disagree |
| 7.溶栓前必须筛查凝血功能。  非常同意 //同意 //中立 //不同意 //非常不同意 | 7. Coagulation profile must be screened before thrombolysis.  Strongly agree //Agree //Neutral //Disagree //Strongly disagree |
| 8.所有急性卒中患者在溶栓前必须进行12导联心电图检查。  非常同意 //同意 //中立 //不同意 //非常不同意 | 8. All acute stroke patients must have a 12 leads ECG before thrombolysis.  Strongly agree //Agree //Neutral //Disagree //Strongly disagree |
| 9.颅内出血是溶栓治疗的禁忌症。  非常同意 //同意 //中立 //不同意 //非常不同意 | 9. Intracranial haemorrhage is a contraindication for thrombolysis therapy.  Strongly agree //Agree //Neutral //Disagree //Strongly disagree |
| **高级卒中管理** | **Advanced stroke management** |
| 1.您是否能够识别急性卒中的症状？  完全可以 //可能可以 //中立 //不太可能 //完全不可能 | 1. Are you able to detect symptoms of acute stroke?  Very likely //Likely //Neutral //Unlikely //Very unlikely |
| 2.您是否熟悉FAST（面部、手臂、言语、时间）或卒中120口诀？  非常熟悉 //熟悉 //中立 //不熟悉 //从未听说过 | 2. Are you familiar with FAST (Face, Arm, Speech, Time)?  Very familiar //Familiar //Neutral //Not familiar //Never heard of it |
| 3.您如何评价您对急性卒中管理的了解？  非常好 //好 //一般 //差 //非常差 | 3. How would you rate your knowledge on acute stroke management?  Very good //Good //Neutral //Poor //Very poor |
| 4.您是否了解卒中的机械取栓术治疗？  非常了解 //了解 //中立 //不了解 //从未听说过 | 4. Are you aware of mechanical thrombectomy treatment for stroke?  Well aware //Aware //Neutral //Not familiar //Never heard of it |
| 5.机械取栓术可用于急性卒中的血栓清除。  非常同意 //同意 //中立 //不同意 //非常不同意 | 5. Mechanical thrombectomy is administered for clot removal in acute stroke.  Strongly agree //Agree //Neutral //Disagree //Strongly disagree |
| 6.我院可以开展机械取栓术。  非常同意 //同意 //中立 //不同意 //非常不同意 | 6. My hospital is equipped with mechanical thrombectomy service.  Strongly agree //Agree //Neutral //Disagree //Strongly disagree |
| 7.急性卒中症状可能可通过溶栓或机械取栓术治疗而恢复。  非常同意 //同意 //中立 //不同意 //非常不同意 | 1. Acute stroke symptoms can be potentially reversed with administration of thrombolysis or with mechanical thrombectomy.   Strongly agree //Agree //Neutral //Disagree //Strongly disagree |
| 8.溶栓治疗后可行机械取栓术。  非常同意 //同意 //中立 //不同意 //非常不同意 | 8. Mechanical thrombectomy can be performed after thrombolysis therapy.  Strongly agree //Agree //Neutral //Disagree //Strongly disagree |
| 9.溶栓治疗和机械血栓切除术只能在各自治疗时间窗内进行。  非常同意 //同意 //中立 //不同意 //非常不同意 | 9. Thrombolysis and mechanical thrombectomy can only be administered within a therapeutic window.  Strongly agree //Agree //Neutral //Disagree //Strongly disagree |
| 1. 醒后卒中不适合溶栓或机械血栓切除术。   非常同意 //同意 //中立 //不同意 //非常不同意 | 10. Wake up strokes are not eligible for thrombolysis normechanical thrombectomy.  Strongly agree //Agree //Neutral //Disagree //Strongly disagree |

**说明：**ASMaQ是一项用于调查医疗保健人员对急性脑卒中管理相关知识了解程度的问卷，共29个条，包含三个子量表：卒中的一般知识（第1-10题）、超急性期卒中管理（第11-19题）和高级卒中管理（第20-29题）。问卷采用1至5分的5级李克特量表，其中7个项目采用反向计分法（第8-11、17、18、29题）。总分范围为29至145分，得分越接近145分表明卒中知识水平越高。(Instruction: The ASMaQ scale was developed to assess healthcare professionals' knowledge level in acute stroke management. The scale comprises 29 items divided into three subscales: General Stroke Knowledge (GSK) (Items 1-10), Hyperacute Stroke Management (HSM) (Items 11-19), and Advanced Stroke Management (ASM) (Items 20-29). The questionnaire uses a 5-point Likert scale for scoring, with 7 items employing reverse scoring (Items 8-11,17,18,29). The total score ranges from 29 to 145, with higher scores closer to 145 indicating greater stroke knowledge.)
