# Supplementary material for: Evaluating the knowledge of stroke management among the non-neurological healthcare professionals in an underdeveloped county in Southwestern China
Source: PLoS One. 2026 Jun 17;21(6):e0351499. doi: 10.1371/journal.pone.0351499 (PMC13274870; doi:10.1371/journal.pone.0351499)
Supplement: S3 Table — (DOCX) [file pone.0351499.s003.docx]

**Supplementary Table S3. Participants’ response to ASMaQ.**

| ASMaQ | Desired response | | Neutral responses | | Undesired response | |
| --- | --- | --- | --- | --- | --- | --- |
|  | Number | Ratio | Number | Ratio | Number | Ratio |
| **General Stroke Knowledge** | | | | | | |
| GSK-1 | 213 | 94.7% | 10 | 4.4% | 2 | 0.9% |
| GSK-2 | 190 | 84.4% | 20 | 8.9% | 15 | 6.7% |
| GSK-3 | 215 | 95.6% | 7 | 3.1% | 3 | 1.3% |
| GSK-4 | 220 | 97.8% | 2 | 0.9% | 3 | 1.3% |
| GSK-5 | 216 | 96.0% | 6 | 2.7% | 3 | 1.3% |
| GSK-6 | 217 | 96.4% | 5 | 2.2% | 3 | 1.3% |
| GSK-7 | 219 | 97.3% | 6 | 2.7% | 0 | 0.0% |
| GSK-8 | 69 | 30.7% | 6 | 2.7% | 150 | 66.7% |
| GSK-9 | 97 | 43.1% | 35 | 15.6% | 93 | 41.3% |
| GSK-10 | 2 | 0.9% | 4 | 1.8% | 219 | 97.3% |
| **Hyperacute Stroke Management** | | | | | | |
| HSM-1 | 75 | 33.3% | 25 | 11.1% | 125 | 55.6% |
| HSM-2 | 200 | 88.9% | 15 | 6.7% | 10 | 4.4% |
| HSM-3 | 179 | 79.6% | 23 | 10.2% | 23 | 10.2% |
| HSM-4 | 219 | 97.3% | 3 | 1.3% | 3 | 1.3% |
| HSM-5 | 213 | 94.7% | 9 | 4.0% | 3 | 1.3% |
| HSM-6 | 187 | 83.1% | 7 | 3.1% | 31 | 13.8% |
| HSM-7 | 0 | 0.0% | 2 | 0.9% | 223 | 99.1% |
| HSM-8 | 1 | 0.4% | 11 | 4.9% | 213 | 94.7% |
| HSM-9 | 216 | 96.0% | 8 | 3.6% | 1 | 0.4% |
| **Advanced Stroke Management** | | | | | | |
| AMS-1 | 199 | 88.1% | 21 | 9.3% | 6 | 2.7% |
| AMS-2 | 142 | 63.1% | 34 | 15.1% | 49 | 21.8% |
| AMS-3 | 144 | 64.0% | 75 | 33.3% | 6 | 2.7% |
| AMS-4 | 136 | 60.4% | 39 | 17.3% | 50 | 22.2% |
| AMS-5 | 202 | 89.8% | 23 | 10.2% | 0 | 0.0% |
| AMS-6 | 159 | 70.7% | 31 | 13.8% | 35 | 15.6% |
| AMS-7 | 209 | 92.9% | 14 | 6.2% | 2 | 0.9% |
| AMS-8 | 174 | 77.3% | 32 | 14.2% | 19 | 8.4% |
| AMS-9 | 175 | 77.8% | 34 | 15.1% | 16 | 7.1% |
| AMS-10 | 39 | 17.3% | 63 | 28.0% | 123 | 54.7% |
