# Supplementary material for: Evaluating the knowledge of stroke management among the non-neurological healthcare professionals in an underdeveloped county in Southwestern China
Source: PLoS One. 2026 Jun 17;21(6):e0351499. doi: 10.1371/journal.pone.0351499 (PMC13274870; doi:10.1371/journal.pone.0351499)
Supplement: S4 Table — (DOCX) [file pone.0351499.s004.docx]

**Supplementary Table S4. The correlation between** **ASMaQ score/subscore and demographic variables in healthcare professionals.**

| Variables | ASMaQ total score | | GSK score | | HSM score | | ASM score | |
| --- | --- | --- | --- | --- | --- | --- | --- | --- |
|  | r | *P* | r | *P* | r | *P* | r | *P* |
| Age | 0.12 | 0.08 | -0.04 | 0.59 | 0.06 | 0.40 | **0.17** | **0.01** |
| Years of work experience | 0.07 | 0.28 | -0.07 | 0.30 | 0.04 | 0.51 | 0.12 | 0.06 |
