# Supplementary material for: Evaluating the knowledge of stroke management among the non-neurological healthcare professionals in an underdeveloped county in Southwestern China
Source: PLoS One. 2026 Jun 17;21(6):e0351499. doi: 10.1371/journal.pone.0351499 (PMC13274870; doi:10.1371/journal.pone.0351499)
Supplement: S5 Table — (DOCX) [file pone.0351499.s005.docx]

**Supplementary Table S5. The comparisons of ASMaQ score/subscore among the healthcare professionals subgroups distributed by gender, education level, job position, professional level, department, medical facility level, and presence of a neurologist partner or not.**

|  | ASmaQ total score, mean [SD] (range) | *P* | GSK score, mean [SD] (range) | *P* | HSM score, mean [SD] (range) | *P* | ASM score, mean [SD] (range) | *P* |
| --- | --- | --- | --- | --- | --- | --- | --- | --- |
| **Gender** |  | **0.003** |  | 0.36 |  | **0.001** |  | **<0.001** |
| Female | 106.7  [7.7] (86-129) |  | 37.9  [2.8] (28-46) |  | 31.3 [2.7] (24-39) |  | 37.5  [4.9] (27-46) |  |
| Male | 110.2 [8.1] (89-125) |  | 37.5 [3.0] (28-46) |  | 32.5  [2.5]  (26-38) |  | 40.2 [4.9] (28-50) |  |
| **Education level** |  | 0.87 |  | 0.67 |  | 0.48 |  | 0.99 |
| Senior high school | 108.2 [8.6] (101-129) |  | 37.9 [3.7] (33-46) |  | 31.8 [3.4] (26-37) |  | 38.5 [4.5] (33-46) |  |
| Associate college | 107.1 [7.5] (89-120) |  | 37.7 [2.9] (28-42) |  | 31.2 [2.8] (24-36) |  | 38.2 [4.7] (28-46) |  |
| Bachelor | 108 [8.2] (86-125) |  | 37.7 [2.8] (28-46) |  | 32 [2.6] (25-39) |  | 38.3 [5.3] (27-50) |  |
| Master/Doctor | 109.5 [8.7] (98-119) |  | 39.8 [3.1] (37-44) |  | 32 [2.9] (29-35) |  | 37.8 [6.2] (31-46) |  |
| **Job position** |  | **<0.001** |  | 0.16 |  | **0.002** |  | **0.001** |
| Doctor | 110 [7.9] (89-125) |  | 38.1 [2.8] (28-44) |  | 32.3 [2.9] (24-39) |  | 39.7 [4.9] (28-50) |  |
| Nurse | 106.3 [7.6] (86-129) |  | 37.6 [2.9] (28-46) |  | 31.3 [2.6] (24-37) |  | 37.4 [5.0] (27-46) |  |
| **Professional level** |  | 0.05 |  | 0.17 |  | 0.19 |  | **0.02** |
| Not yet rated | 104.7 [6.8] (92-115) |  | 38.2 [2.9] (31-42) |  | 30.9 [3.2] (24-36) |  | 35.6 [4.7] (27-42) |  |
| Junior | 107 [8.2] (86-129) |  | 37.5 [3.0] (28-46) |  | 31.5 [2.9] (24-39) |  | 38 [5.0] (27-46) |  |
| Intermediate | 109.3 [7.4] (89-125) |  | 38.1 [2.6] (30-44) |  | 31.9 [2.3] (25-36) |  | 39.3 [4.9] (29-50) |  |
| Senior | 108.6 [8.5] (89-123) |  | 37.1 [3.3] (28-42) |  | 32.5 [2.5] (27-38) |  | 39.1 [5.2] (32-47) |  |
| **Department** |  | 0.69 |  | 0.74 |  | 0.68 |  | 0.35 |
| Internal Medicine | 108.0  [7.8] (92-129) |  | 37.6  [3.1] (32-46) |  | 32.3  [2.3] (28-37) |  | 38.1  [4.9] (27-46) |  |
| Surgery | 108.6  [7.4] (91-123) |  | 38.0  [2.5] (30-44) |  | 32.1  [2.8] (24-39) |  | 38.5  [4.9] (28-47) |  |
| Emergency and ICU | 110.0  [5.6] 102-125) |  | 37.7  [2.7] (31-43) |  | 32.2  [1.8] (29-36) |  | 40.0  [4.0] (31-50) |  |
| Others | 108.8  [7.2] (97-115) |  | 38.2  [2.2] (34-40) |  | 33.3  [2.3] (31-36) |  | 37.3  [4.8] (32-44) |  |
| Undifferentiated | 105.2 [37.7] (30.0-37.4) |  | 9.2 [3.2] (2.9-5.8) |  | 120 [42] (36-46) |  | 34 [14] (12-19) |  |
| **Medical facility level** |  | **0.02** |  | 0.83 |  | **<0.001** |  | 0.16 |
| Comprehensive hospital | 108.5 [7.3] (91-129) |  | 37.8 [2.8] (30-46) |  | 32.2 [2.4] (24-39) |  | 38.6 [4.8] (27-50) |  |
| Non-comprehensive medical facilities | 105.2 [9.3] (86-120) |  | 37.7 [3.2] (28-42) |  | 30.04 [2.9] (24-36) |  | 37.5 [5.8] (27-46) |  |
| **With neurologists partner in the routine clinical practise** |  | **0.005** |  | 0.25 |  | 0.11 |  | **0.01** |
| Yes | 109 [7.5] (86-125) |  | 38 [2.8] (28-46) |  | 32 [2.4] (25-39) |  | 39.1 [4.9] (28-50) |  |
| No | 106.1 [8.1] (89-129) |  | 37.4 [2.9] (28-46) |  | 31.3 [3.1] (24-37) |  | 37.4 [5.1] (27-46) |  |
